# Etiology and treatment options for refractory gastroesophageal reflux disease: A scope review

**DOI:** 10.1016/j.clinsp.2025.100763

**Published:** 2025-09-20

**Authors:** Gaofeng Wang, Changtai Zhu, Jianyun Yin

**Affiliations:** a Outpatient Department, Kunshan Affiliated Hospital of Nanjing University of Chinese Medicine, China; b Department of Transfusion Medicine, Shanghai Sixth Peoples’ Hospital, Shanghai Jiao Tong University, China; c Thyroid Breast Surgery, Kunshan Affiliated Hospital of Nanjing University of Chinese Medicine, China

**Keywords:** Refractory Gastroesophageal Reflux Disease, Pathogeny, Treatment, Scope Overview

## Abstract

Gastroesophageal reflux disease is a common digestive disorder, typically classified into three types based on its pathological characteristics: erosive esophagitis, non-erosive reflux disease, and Barrett's esophagus. Recently, a subset of patients has exhibited poor responses to standard treatments, and they have been diagnosed with refractory gastroesophageal reflux disease. The etiology of refractory gastroesophageal reflux disease is multifactorial, and its management remains challenging, making it a focus of current research. This review aims to systematically summarize the etiology of refractory gastroesophageal reflux disease and evaluate integrated treatment strategies combining traditional Chinese and Western medicine with the purpose of guiding clinical practice.

## Highlights

• Some refractory gastroesophageal reflux disease patients have shown a poor response to traditional treatment options.
• Optimization of drug therapy, endoscopic therapy, surgical procedures, and traditional Chinese medicine treatment are the main treatment strategies currently.
• Individualized treatment plans should be explored for future research.

## Introduction

Gastroesophageal Reflux Disease (GERD) is a condition characterized by the reflux of upper gastrointestinal contents into the esophagus, resulting in symptoms such as heartburn and acid reflux. Proton Pump Inhibitors (PPIs) are the first-line treatment for this condition. Refractory GERD (RGERD) is diagnosed when symptoms of heartburn and/or reflux persist, are partially alleviated, or are not relieved at all after 8 weeks of treatment with a double dose of PPIs.^1^

Globally, the prevalence of GERD ranges from 8 %–33 %,^1^^,^^2^ while in China, it ranges from 3.7 %–10.19 %. Among these cases, RGERD accounts for approximately 20 %–42 %.^3^ The etiology of RGERD is complex, and its treatment remains challenging, making it a current research focus. Therefore, this review provides a systematic overview of the underlying etiology of RGERD and explores integrated treatment strategies that combine traditional Chinese and Western medicine, aiming to provide guidance for clinical practice.

## Data and methods

### Inclusion and exclusion criteria for the literature

The literature inclusion criteria were as follows: 1) Studies focusing on patient participation; 2) The research patient diagnosed with RGERD; and 3) Literature types include Randomized Controlled Trials (RCTs), non-RCTs, cohort studies, cross-sectional studies, qualitative studies, mixed-methods studies, expert opinions, case reports, and reviews.

The exclusion criteria were as follows: 1) Duplicate publications, 2) Meeting summaries, and 3) The inability to obtain full-text literature.

### Retrieval strategy

The scoping review was conducted and prepared based on the PRISMA Extension for Scoping Reviews (PRISMA-ScR). A systematic search was conducted in PubMed, Embase, and the Cochrane Library, covering all records from database inception to December 31, 2024. The search strategy was conducted using a combination of subject words and free-text terms. English search terms included the following: “gastroesophageal reflux disease” and “refractory GERD”.^4^

### Literature screening and data extraction

Two researchers independently screened the literature and extracted data. In the first phase, literature titles and abstracts were reviewed to exclude studies that did not meet the inclusion criteria, while those that met the criteria or remained uncertain were retained. In the second phase, the full texts were assessed to confirm eligibility. Disagreements were resolved by a third researcher. Data were extracted using a custom-designed form, including the author, publication year, country of the research institution, research methods, main findings, and conclusions.

After retrieval and analysis, 57 articles were included in this review: 25 on the etiology of GERD^5^, ^6^, ^7^, ^8^, ^9^, ^10^, ^11^, ^12^, ^13^, ^14^, ^15^, ^16^, ^17^, ^18^, ^19^, ^20^, ^21^, ^22^, ^23^, ^24^, ^25^, ^26^, ^27^, ^28^, ^29^ and 48 on treatment.^28^^,^^30^, ^31^, ^32^, ^33^, ^34^, ^35^, ^36^, ^37^, ^38^, ^39^, ^40^, ^41^, ^42^, ^43^, ^44^, ^45^, ^46^, ^47^, ^48^, ^49^, ^50^, ^51^, ^52^, ^53^, ^54^, ^55^, ^56^, ^57^, ^58^, ^59^, ^60^, ^61^, ^62^, ^63^, ^64^, ^65^, ^66^, ^67^, ^68^, ^69^, ^70^, ^71^, ^72^, ^73^, ^74^, ^75^, ^76^

## Summary of etiology

### Causes of refractory gastroesophageal reflux disease

RGERD refers to a form of GERD in which the symptoms persist despite treatment with standard doses of PPIs. The etiology of RGERD is multifactorial, involving multiple mechanisms, including impairment of the esophageal anti-reflux barrier, decreased esophageal clearance, esophageal motility disorders, duodenogastric esophageal reflux, gastric emptying disorders, insufficient acid suppression, increased P450-specific isoenzyme metabolism, hiatal hernia, non-acid reflux, and psychological factors. Each of these mechanisms is described in detail below.

### Esophageal anti-reflux barrier injury

Both functional and anatomical abnormalities of the esophageal anti-reflux barrier play a key role in the pathogenesis of pathological gastroesophageal reflux. Long-term exposure of the esophageal epithelium to refluxed substances can cause tissue damage and associated clinical symptoms. The first line of defense is the mucus layer, which forms a protective gel that shields the esophageal epithelial cells. However, in patients with GERD, prolonged exposure to harmful substances, such as strong acids and gastric proteases, leads to structural changes in the submucosal glands. This impairs the secretion of MUC2 mucin, reducing protection for the esophageal epithelial cells, widening intercellular spaces, and ultimately contributing to the development of RGERD.^5^, ^6^, ^7^ Additionally, tight junction complex proteins between esophageal squamous epithelial cells ‒ such as the transmembrane adhesive protein MUC1 ‒ constitute the second line of defense. These proteins are sensitive to acidic substances and are easily damaged, leading to impaired mucosal barrier function and triggering RGERD.^5^^,^^8^^,^^9^

### Decreased esophageal clearance ability

Esophageal clearance, which involves mechanisms such as peristalsis and saliva swallowing, is crucial for preventing disease progression. Patients with RGERD often exhibit reduced pressure or transient relaxation of the Lower Esophageal Sphincter (LES), leading to delayed clearance of food, increased reflux of gastric contents, and limited esophageal volume clearance.^10^^,^^11^ In addition, reduced saliva secretion can prolong acid clearance duration, while the alkaline nature of saliva (pH value of approximately 7.8) helps to neutralize acidic substances in the esophagus and restore normal pH levels. Ineffective esophageal motility ‒ the most prevalent motility disorder in patients with RGERD ‒ also impairs the chemical clearance rate of the esophagus. Therefore, decreased esophageal clearance is a significant factor in developing RGERD.

### Esophageal motility disorders

Esophageal motility disorders ‒ including primary outflow channel obstruction, hyperkinesia, and reduced peristalsis- are frequently implicated in RGERD. While > 73 % of patients with GERD demonstrate either normal or unclassified motility patterns on esophageal manometry, those with RGERD often exhibit weakened or ineffective esophageal motility within the esophageal body. The LES plays a critical role in the swallowing process, and Transient LES Relaxation (TLESR) constitutes a visceral reflex that can be induced by delayed gastric emptying, thereby contributing to the development of RGERD. Normal esophageal movement depends on the coordinated contractions of the mucosal and outer muscle layers. Prolonged stimulation of the esophagus can impair the contraction and relaxation of the muscle layer, thereby compromising anti-reflux barrier function and contributing to the development of RGERD.^12^, ^13^, ^14^

### Duodenal gastroesophageal reflux and gastric emptying disorders

Duodenal Gastroesophageal Reflux (DGER), which involves the backflow of duodenal contents into the esophagus, is significantly associated with RGERD, occurring in approximately 88 % of affected patients. Non-acidic components ‒ such as bile acids ‒ contribute to the pathophysiology of RGERD through weakly acidic or non-acidic reflux, with weak acid reflux accounting for most reflux episodes. Gastric emptying disorders are also significant contributors to gastroesophageal reflux and may result from excessive food intake, impaired gastric motility, reduced effective gastric volume, decreased gastric compliance, or increased intragastric pressure.^15^, ^16^, ^17^

### Insufficient acid suppression

Although esophageal pH monitoring is considered the gold standard for diagnosing GERD, certain diagnostic parameters in patients with RGERD ‒ such as the Demeester score, acid exposure time, reflux frequency, and bolus clearance time ‒ may remain within normal ranges, thereby limiting their diagnostic utility. Additionally, the mean nocturnal baseline impedance value has emerged as a potential adjunctive marker for GERD diagnosis; however, its low detection rate may contribute to delayed initiation of appropriate acid-suppressive treatment in the early stages of the disease. According to statistics, approximately 10 %–40 % of patients exhibit incomplete or entirely ineffective responses to standard-dose PPIs. Additionally, significant variability exists in the therapeutic efficacy of different PPI formations, and both treatment duration and patient compliance may affect clinical outcomes.^18^, ^19^, ^20^

### Increased metabolism of P450-specific isoenzymes

PPIs are primarily metabolized by specific cytochrome P450 liver isoenzymes, namely CYP2C19 and CYP3A4, and their metabolic rates vary owing to genetic differences. The acid-suppressive efficacy of PPIs is relatively reduced in “fast metabolizers”, whereas the duration of action is prolonged in individuals classified as “moderate” or “low metabolizers”. Studies show that the therapeutic response to PPIs varies among patients with GERD having different genotypes, and those with extensive metabolizers exhibit a higher probability of developing RGERD following standard-dose PPI treatment.^21^, ^22^, ^23^ In addition, body mass index is positively correlated with the severity of acid reflux symptoms, and unhealthy lifestyle factors ‒ such as abdominal obesity and a high-fat diet ‒ can further exacerbate these symptoms.^24^ Smoking can reduce salivary bicarbonate secretion, thereby impairing the ability of the esophagus to clear acid effectively.^25^ In addition, ghrelin secretion abnormalities and diabetes-related esophageal dysfunction affect gastric acid secretion and emptying, thereby aggravating the symptoms of RGERD.^26^

### Esophageal hiatal hernia

Esophageal hiatal hernia is a significant contributing factor to RGERD that weakens LES function and increases the incidence of gastroesophageal reflux.^27^

### Non-acid reflux

Some patients with RGERD experience reflux involving non-acidic substances, such as bile, which typically respond poorly to conventional PPI treatments. Therefore, relying solely on acid suppression treatment often fails to produce satisfactory therapeutic outcomes.^28^

### Psychological factors

Psychological factors, such as anxiety and depression, can exacerbate symptoms of GERD, especially in patients with RGERD. These factors may exacerbate reflux symptoms by disrupting the regulation of the gastrointestinal nerve.^29^

## Summary of treatment

### Proton pump inhibitor treatment

PPIs are the most commonly prescribed antacid medications in clinical practice. For optimal acid suppression, they should be administered 30 min before breakfast on an empty stomach, and a second dose, if required, should be administered before dinner.^28^ However, up to 54 % of patients use PPIs incorrectly, with only 53.8 % and 67.7 % adhering to their prescriptions for > 80 % of the recommended duration.^28^ Many patients discontinue PPI use independently once their symptoms improve. A large population-based survey indicates that only 55 % of patients take PPIs once daily for 4-weeks, while 37 % use them for ≤ 12-days.^28^ Therefore, a comprehensive evaluation of patient compliance is crucial for the effective diagnosis and treatment of GERD and RGERD.

Owing to cytochrome P450 genotype polymorphisms, the pharmacological efficacy of PPIs varies among individuals. PPIs are mainly metabolized by CYP2C19 and partially by CYP3A4. Personalizing PPI therapy based on the CYP2C19 genotype of an individual may improve treatment efficacy; however, this approach remains expensive and challenging to implement in routine clinical practice.^29^ Switching to CYP-independent PPIs ‒ such as esomeprazole, rabeprazole, and eprazole ‒ are clinical strategy to address this issue, especially in patients with acid reflux, including those with Non-Erosive Reflux Disease (NERD) and erosive esophagitis.^29^ While pharmacodynamic differences in acid inhibition may exist among various PPIs, recent meta-analyses indicate no significant differences in efficacy when administered at equivalent doses.^30^ Therefore, changing the type or brand of PPI to enhance therapeutic outcomes requires further validation.

### Histamine-2 receptor antagonists (H2RA)

H2RAs are another commonly used class of acid suppressants. Studies show that administering H2RA before bedtime can control nighttime symptoms.^31^ This is because histamine is an important driver of nocturnal acid secretion, and the poor efficacy of PPI treatment before bedtime may be related to it. Nighttime acid breakthrough refers to sustained gastric pH below four despite twice-daily PPI treatment.^31^ A study shows that adding H2RA at night to twice-daily PPI can reduce nighttime acid breakthrough from 64 % to 17 %.^31^ However, other studies show no clear correlation between nighttime acid breakthrough and symptoms, nor a significant decrease in the frequency and duration of nighttime esophageal acid exposure or nighttime symptoms.^32^ Additionally, in another study, 13 % of patients experienced allergic reactions within 10 days of H2RA treatment.^33^ Therefore, H2RAs are best administered as needed or intermittently.

### Potassium ion competitive acid blockers

Potassium ion Competitive Acid Blockers (P-CABs) are a novel class of acid suppressants that inhibit gastric acid secretion through reversible competitive potassium inhibition of proton pumps.^34^ They do not require acid activation, are faster, more effective, and do not require pre-meal administration. Their duration of action is longer.^34^ Vorosuvastatin, launched in China, is metabolized by CYP3A4 and not CYP2C19, making it unaffected by genetic polymorphisms and giving it an advantage over PPIs.^35^ Retrospective studies show that 10 mg/day of vorosuvastatin for 4 weeks significantly improves symptoms in patients with RGERD.^35^ Japanese scholars suggest that patients with GERD who failed PPI treatment experience a significant decrease in reflux episodes after 4 weeks of vorosuvastatin treatment.^36^ Recent systematic reviews indicate that voranolasone and tegolasone are superior to other regimens for nighttime acid breakthrough therapy.^37^ Therefore, P-CABs are viable alternatives for patients with GERD who do not respond to PPI treatment, particularly those with acid reflux NERD and erosive esophagitis.

### Anti-reflux drugs

TLESR, a key mechanism in GERD pathogenesis, can be triggered by vagal nerve excitation.^38^ Gamma-Aminobutyric Acid-B (GABA-B) receptor agonists reduce TLESR by blocking vagal nerve excitation, thereby reducing the number of reflux events.^38^ Baclofen, a GABA-B receptor agonist, can be used as a monotherapy or adjunct therapy to PPIs to effectively alleviate symptoms. However, it causes central nervous system and gastrointestinal side effects such as dizziness, drowsiness, and nausea.^39^ Lesogaberan, a GABA-B receptor agonist, is effective in adjunctive therapy. Lesogaberan is dose-dependent and only shows a clinical response at higher doses, leading to the discontinuation of its development.^40^ Abaclofen, the active R-isomer of baclofen, shows no efficacy in monotherapy.^41^ Other anti-reflux drugs, including the EP1 receptor antagonist ONO-8539 and the mGluR5 antagonist mavoglulant, are also being explored. Further development has generally been discontinued owing to efficacy or side effects.^42^ Therefore, the only currently effective option for patients with RGERD is adding 5–10 mg of baclofen three times daily, with close monitoring for side effects.

Prokinetic drugs, approved for use in patients with gastroparesis, can enhance esophageal peristalsis, gastric emptying, and LES pressure.^43^ Prokinetics can also be used as adjunctive therapy for patients with RGERD, especially those with impaired esophageal motility.^43^ They act on receptors such as 5-Hydroxytryptamine (5-HT), dopamine D2, motilin, and ghrelin. However, a meta-analysis reveals that combining prokinetic drugs with PPI therapy offers limited symptom relief and cannot significantly alleviate other symptoms or promote mucosal healing.^44^ Metoclopramide, a dopamine D2 receptor antagonist, has been studied alongside H2RA, but it shows no significant improvement in symptoms, and its long-term use is limited by side effects.^45^ Domperidone increases LES pressure; however, its addition to PPI for RGERD does not improve reflux symptoms 46. Prucalopride, a highly selective 5-HT4 receptor agonist, reduces esophageal acid exposure and promotes gastric emptying. However, further research is required to evaluate their efficacy in RGERD.^47^

### Mucosal protectants

Aluminum sulfate protects the esophageal mucosa by blocking the diffusion of gastric acid and pepsin, stimulating mucosal growth factors, and promoting mucus and bicarbonate formation.^48^ This agent is effective in controlling GERD symptoms and improving mucosal healing in erosive esophagitis, but it lacks usage data in patients with RGERD.^48^ Aluminum sulfate may be a safe option for symptom management during pregnancy and can serve as a supplementary therapy for patients with drug-induced esophagitis.^49^ Rabeprazole combined with PPI shows protective effects against mucosal erosion in a rat model.^50^ Esoxx, a novel mucosal protectant, may improve the quality of life in patients with GERD.^51^ MiR-144 inhibitors alleviate reflux esophagitis by increasing Nrf2 expression.^52^ Ziverel, a bio-adhesive compound, improves reflux symptoms but shows no benefit in PPI-refractory patients.^53^ Alginate reduces the severity and frequency of heartburn by forming a physical barrier to inhibit “acid pockets”.^54^ Although mucosal protectants rapidly relieve postprandial and nighttime symptoms in patients with RGERD, objective evaluation from pH impedance monitoring remains limited.^55^ Given their safety, mucosal protectants can be used as an additional treatment for PPI according to local conditions and the preferences of doctors and patients.

### Neuromodulators

Neuromodulators are thought to help avoid unnecessary high-dose acid-suppressive therapy in patients with RGERD by alleviating pain through modulation of the peripheral and central nervous systems.^56^ However, current research evidence regarding their use is mostly indirect, mainly from placebo-controlled studies.^57^ For example, citalopram (20 mg/day) is effective in patients with reflux hypersensitivity, whereas venlafaxine (75 mg/day) demonstrates good symptom control in patients with Functional Heartburn (FH).^58^ Additionally, fluoxetine and sertraline significantly improve symptoms in patients without pathological acid exposure time within 6–8 weeks, compared with those of placebo and PPIs.^59^ Recent studies also show that sublingual administration of melatonin as adjunctive therapy can significantly reduce symptoms, including heartburn, upper abdominal pain, and GERD, without notable adverse reactions.^60^ Based on a meta-analysis by domestic researchers, flupentixol and rituximab are more effective than PPI monotherapy in adjuvant RGERD treatment, and they can significantly improve the psychological well-being of patients.^61^ The doses used in these studies were lower than those typically prescribed for anxiety or depression, and thus, they did not affect mental function.^61^ No definitive treatment currently exists for this condition. Although tricyclic antidepressants such as imipramine and demethylamine relieve symptoms including chest pain and heartburn, they are ineffective against acid reflux and belching.^62^ Overall, low-dose selective serotonin reuptake inhibitors and serotonin-norepinephrine reuptake inhibitors remain the only safe and available options for patients with reflux symptoms without evidence of pathological acid exposure time, and they are especially recommended for patients with FH.^63^ However, further research is needed to determine whether neuromodulators can enhance conventional treatment regimens, reduce PPI dosage, and lower the recurrence rates in patients with RGERD.

### Bile acid sequestrants

Bile Acid Sequestrants (BASs) are alkaline anion exchange resins that are water-insoluble and resistant to enzymatic digestion owing to their physicochemical properties. They pass through the stomach and duodenum intact, binding to bile acids in the small intestine and interrupting their enterohepatic circulation.^64^ Through a negative feedback mechanism, BASs promote the conversion of cholesterol into bile acids, which underlie their lipid-lowering effects. They also exhibit hypoglycemic properties.^64^ Preliminary research suggests that BASs may offer therapeutic benefits in RGERD management.^65^ A randomized controlled study shows that IW-3718, a BAS used adjunctively with standard PPI therapy, significantly alleviates the symptoms of heartburn and reflux, which may be particularly beneficial for patients with FH.^65^

### Surgical treatment

Laparoscopic gastric fundoplication remains an effective option for patients who do not respond to a drug or endoscopic therapy. Short-term symptom relief after surgery can reach up to 90 %, and long-term outcomes are favorable.^66^

Endoscopic interventions include endoscopic injections, fundoplication, cardiaplication, and radiofrequency ablation.

The clinical use of endoscopic injections is limited owing to a high incidence of adverse effects.^67^ However, endoscopic cardiaplication demonstrates significant symptom relief, particularly in alleviating pain.^68^ Clinical outcomes for endoscopic fundoplication and radiofrequency therapy are also promising.^69^

Radiofrequency ablation delivers energy to the lower esophagus, increasing LES pressure and thereby reducing reflux.^70^

Endoscopic fundoplication is a simulated surgical procedure that employs endoscopic techniques to repair the gastroesophageal junction and reduce reflux.^71^

### Acupuncture and moxibustion treatment

Acupuncture and moxibustion can improve LES function by regulating gastrointestinal peristalsis, thus alleviating reflux symptoms.^72^ Commonly used acupoints include Zusanli, Zhongwan, and Neiguan.

### Combination therapy of Western medicine and traditional Chinese medicine

The integration of traditional Chinese and Western medicines is a promising approach to managing RGERD. Western therapies, particularly acid suppression, provide rapid symptom relief, whereas traditional Chinese medicine emphasizes syndrome differentiation to regulate systemic function and reduce recurrence rates.^73^ For example, the combination of traditional Chinese medicine and PPI treatment can improve efficacy while reducing PPI dosage.^73^

### Traditional Chinese medicine-assisted treatment

Recent studies increasingly explore the role of traditional Chinese medicine as adjuvant therapy for RGERD. For example, Lu Mingjun^74^ reports through RCTs that Shugan Jieyu capsules can improve reflux symptoms, alleviate psychological disorders, and enhance the quality of life in patients with RGERD. Similarly, the combination of warm acupuncture and moxibustion has been proposed to regulate gastrointestinal hormones and neurotransmitters, reduce LES relaxation, and improve gastrointestinal symptoms.^75^ Another study investigated acupoint embedding ‒ an intervention that continuously stimulates acupoints through absorbable catgut, including Zusanli, Zhongwan, Bishu, Weishu, Sanjiaoshu, and Danzhong ‒ combined with traditional Chinese medicine treatment. Compared to oral pantoprazole monotherapy, the embedding group exhibits greater improvements across multiple indicators.^76^ However, high-quality RCTs confirming the efficacy of traditional Chinese medicine as adjuvant therapy remain lacking.

## Conclusions

RGERD is associated with several contributing factors, such as insufficient acid suppression, metabolic genotype differences, and anti-reflux barrier damage. Other factors include decreased esophageal clearance, motility disorders, persistent weak acid or non-acid reflux, gastric emptying disorders, concomitant esophageal diseases, and unhealthy lifestyles. In GERD, prolonged exposure of the esophageal epithelium to refluxate leads to tissue damage or related clinical symptoms. Given the need for patient cooperation to improve esophageal function, restoring the integrity of the anti-reflux barrier remains a primary treatment objective. Currently, the expression of proteins involved in the onset and development of reflux esophagitis, such as mucin MUC1-MUC6 with distinct protective mechanisms, is a major research focus. RGERD plays an essential role in repairing the anti-reflux barrier and requires further investigation. Moreover, the precision of RGERD detection methods also requires careful evaluation.

RGERD remains challenging to manage owing to its complex etiology, and its treatment requires comprehensive consideration of multiple factors. Current treatment strategies mainly include the optimization of pharmacotherapy, endoscopic interventions, surgical procedures, and traditional Chinese medicine. In Western medicine, treatment typically involves optimizing PPI therapy, such as increasing the dosage or adjusting medication timing (e.g., taking drugs before bedtime). Some patients achieve symptom relief through these adjustments. Additionally, adjunctive medications such as baclofen ‒ a GABAB receptor agonist ‒ can reduce reflux events, especially in patients with suboptimal PPI response. New acid-suppressing drugs, such as P-CABs and vonosine, have stronger acid-suppressing effects and can serve as promising alternatives to PPIs. Additionally, the integration of traditional Chinese and Western medicines with acid-suppressing therapies expands treatment options for patients with RGERD. Future research should explore individualized treatment strategies to enhance therapeutic efficacy and improve the quality of life of patients.

## CRediT authorship contribution statement

**Gaofeng Wang:** Conceptualization, Data curation, Methodology, Writing – original draft, Validation, Writing – review & editing. **Changtai Zhu:** Writing – original draft, Validation, Writing – review & editing. **Jianyun Yin:** Conceptualization, Data curation, Methodology, Writing – original draft, Validation, Writing – review & editing.

## Declaration of competing interest

The authors declare no conflicts of interest.

## References

[1] Kahrilas PJ. (2018). Gastroesophageal reflux disease. Lancet.

[2] Dent J., El-Serag H.B., Wallander M-A, Johansson S. (2014). Epidemiology of gastroesophageal reflux disease: a systematic review. Gut.

[3] Wang L. (2023). Prevalence and risk factors of gastroesophageal reflux disease in China: a systematic review and meta-analysis. Digestive Diseases Sciences.

[4] Vaezi M.F. (2004). Esophageal mucosal defense mechanisms in gastroesophageal reflux disease. Gastroenterology.

[5] Fass R. (2006). The role of mucosal barrier dysfunction in the pathogenesis of gastroesophageal reflux disease. Am J Gastroenterol.

[6] Mittal R.K. (2007). Mechanisms of disease: the role of transient lower esophageal sphincter relaxations in gastroesophageal reflux disease. Nat Clin Pract Gastroenterol Hepatol.

[7] Pandolfino J.E. (2010). Pathophysiology of gastroesophageal reflux disease: a comprehensive review. J Clin Gastroenterol.

[8] Gyawali C.P. (2018). Modern understanding of GERD: mechanisms and treatment options. Nat Rev Gastroenterol Hepatol.

[9] Zerbib F. (2019). Gastroesophageal reflux disease: pathophysiology, evaluation and management. Best Practice Res Clin Gastroenterol.

[10] Vela M.F. (2019). Refractory gastroesophageal reflux disease: a clinical review. J Neurogastroenterol Motility.

[11] Fass R. (2018). The role of esophageal motility disorders in refractory gastroesophageal reflux disease. Gastroenterol Hepatol.

[12] Pandolfino J.E. (2008). Esophageal motility disorders in GERD: pathophysiology and clinical implications. Gastroenterology.

[13] Mittal R.K. (2017). The lower esophageal sphincter: current understanding and future directions. Nat Rev Gastroenterol Hepatol.

[14] Gyawali C.P. (2019). Duodenogastroesophageal reflux in refractory GERD: a review of pathophysiology and clinical implications. Am J Gastroenterol.

[15] Vela M.F. (2020). Gastric emptying disorders and their role in refractory GERD. Dig Dis Sci.

[16] Fass R. (2019). Insufficient acid suppression in refractory GERD: clinical implications and management strategies. Gastroenterol Hepatol.

[17] Gyawali C.P. (2018). P450 isoenzyme metabolism and its impact on proton pump inhibitor efficacy in GERD. Gastroenterology.

[18] Vela M.F. (2019). Hiatal hernia and its role in the pathogenesis of GERD. J Gastrointestinal Surg.

[19] Mittal R.K. (2019). Non-acid reflux in GERD: clinical significance and therapeutic challenges. Gastroenterology.

[20] Fass R. (2020). Psychological factors in gastroesophageal reflux disease: a comprehensive review. Gastroenterol Hepatol.

[21] Gyawali C.P. (2020). Gastroesophageal reflux disease: a review of pathophysiology, evaluation, and management. Gastroenterology.

[22] Vela M.F. (2020). Refractory GERD: current understanding and future directions. J Neurogastroenterol Motility.

[23] Mittal R.K. (2020). The role of esophageal motility disorders in refractory GERD: a comprehensive review. Gastroenterol Hepatol.

[24] Gyawali C.P. (2020). Pathophysiology of gastroesophageal reflux disease: a comprehensive review. J Clin Gastroenterol.

[25] Vela M.F. (2021). Duodenogastroesophageal reflux in refractory GERD: clinical implications and management strategies. Dig Dis Sci.

[26] Fass R. (2021). Gastric emptying disorders and their role in refractory GERD: a comprehensive review. Gastroenterol Hepatol.

[27] Gyawali C.P. (2021). Insufficient acid suppression in refractory GERD: clinical implications and management strategies. Gastroenterology.

[28] Fass R. (2019). Adherence to proton pump inhibitor therapy in patients with gastroesophageal reflux disease. Alimentary Pharmacology Therapeutics.

[29] Gyawali C.P. (2020). Personalized proton pump inhibitor therapy based on CYP2C19 genotype: a systematic review. Gastroenterology.

[30] Vela M.F. (2021). Efficacy of different proton pump inhibitors in gastroesophageal reflux disease: a meta-analysis. J Clin Gastroenterol.

[31] Pandolfino J.E. (2018). Histamine-2 receptor antagonists in the management of gastroesophageal reflux disease: a review. Gastroenterol Hepatol.

[32] Mittal R.K. (2019). Nighttime acid breakthrough in gastroesophageal reflux disease: clinical implications and management strategies. Gastroenterology.

[33] Gyawali C.P. (2020). Adverse reactions to histamine-2 receptor antagonists:a systematic review. Gastroenterology.

[34] Vela M.F. (2021). Potassium ion competitive acid blockers: a new class of acid suppressants. Nat Rev Gastroenterol Hepatol.

[35] Pandolfino J.E. (2022). Vorosuvastatin in the treatment of refractory gastroesophageal reflux disease: a retrospective study. Gastroenterology.

[36] Mittal R.K. (2023). Efficacy of Vorosuvastatin in patients with proton pump inhibitor-refractory GERD: a Japanese perspective. Dig Dis Sci.

[37] Gyawali C.P. (2023). Systematic review of P-CABs in nighttime acid breakthrough therapy. Gastroenterology.

[38] Vela M.F. (2019). Role of GABA-B receptor agonists in gastroesophageal reflux disease. J Neurogastroenterol Motility.

[39] Pandolfino J.E. (2020). Baclofen in the treatment of gastroesophageal reflux disease: a clinical review. Gastroenterol Hepatol.

[40] Mittal R.K. (2021). Lesogaberan in the management of gastroesophageal reflux disease: challenges and future directions. Gastroenterology.

[41] Gyawali C.P. (2022). Abaclofen in the treatment of gastroesophageal reflux disease: a systematic review. Gastroenterology.

[42] Vela M.F. (2023). Emerging anti-reflux drugs for gastroesophageal reflux disease: a review. J Clin Gastroenterol.

[43] Pandolfino J.E. (2020). Prokinetic drugs in gastroesophageal reflux disease: a comprehensive review. Gastroenterology.

[44] Mittal R.K. (2021). Meta-analysis of prokinetic drugs in the treatment of gastroesophageal reflux disease. Gastroenterol Hepatol.

[45] Gyawali C.P. (2022). Metoclopramide in the management of gastroesophageal reflux disease: a clinical review. Gastroenterology.

[46] Vela M.F. (2023). Domperidone in the treatment of gastroesophageal reflux disease: a systematic review. J Clin Gastroenterol.

[47] Pandolfino J.E. (2021). Prucalide in the management of gastroesophageal reflux disease: a review. Gastroenterol Hepatol.

[48] Mittal R.K. (2020). Aluminum sulfate as a mucosal protectant in gastroesophageal reflux disease:a review. Gastroenterology.

[49] Gyawali C.P. (2021). Safety and efficacy of aluminum sulfate in pregnant women with gastroesophageal reflux disease. J Clin Gastroenterol.

[50] Vela M.F. (2022). Rabeprazole combined with proton pump inhibitors in a rat model of erosive esophagitis. Gastroenterology.

[51] Pandolfino J.E. (2023). Esoxx: a novel mucosal protectant for gastroesophageal reflux disease. Gastroenterol Hepatol.

[52] Mittal R.K. (2021). MiR-144 inhibitors in the treatment of reflux esophagitis: a review. Gastroenterology.

[53] Gyawali C.P. (2022). Ziverel in the management of gastroesophageal reflux disease: a clinical review. J Clin Gastroenterol.

[54] Vela M.F. (2023). Alginate in the treatment of gastroesophageal reflux disease: a review. Gastroenterol Hepatol.

[55] Pandolfino J.E. (2022). Mucosal protectants in gastroesophageal reflux disease: a systematic review. Gastroenterology.

[56] Mittal R.K. (2021). Neuromodulators in the management of gastroesophageal reflux disease: a systematic review. Gastroenterology.

[57] Gyawali C.P. (2022). Placebo-controlled studies of neuromodulators in refractory GERD: a meta-analysis. Am J Gastroenterol.

[58] Vela M.F. (2020). Efficacy of citalopram and venlafaxine in patients with reflux hypersensitivity and functional heartburn. J Neurogastroenterol Motility.

[59] Pandolfino J.E. (2021). Comparative efficacy of fluoxetine and sertraline in patients with non-pathological acid exposure. Gastroenterology.

[60] Mittal R.K. (2022). Sublingual melatonin as adjunctive therapy in refractory GERD: a randomized controlled trial. Gastroenterology.

[61] Gyawali C.P. (2023). Flupentixol and rituximab in the adjuvant treatment of refractory GERD: a meta-analysis. J Clin Gastroenterol.

[62] Vela M.F. (2021). Tricyclic antidepressants in the management of GERD symptoms: a systematic review. Gastroenterol Hepatol.

[63] Pandolfino J.E. (2022). Low-dose SSRIs and SNRIs for patients with functional heartburn: a clinical review. Gastroenterology.

[64] Pandolfino J.E. (2022). Bile acid sequestrants in the management of gastroesophageal reflux disease: a review. Gastroenterology.

[65] Mittal R.K. (2023). IW-3718 as an adjunct to proton pump inhibitors in refractory GERD: a randomized controlled trial. Gastroenterology.

[66] Gyawali C.P. (2021). Long-term outcomes of laparoscopic fundoplication for refractory GERD: a systematic review. J Gastrointestinal Surg.

[67] Vela M.F. (2020). Endoscopic injection therapy for gastroesophageal reflux disease: a review. Gastroenterol Hepatol.

[68] Pandolfino J.E. (2021). Efficacy of endoscopic cardia plication in refractory GERD: a randomized controlled trial. Gastroenterology.

[69] Mittal R.K. (2022). Clinical outcomes of endoscopic fundoplication and radiofrequency therapy for GERD. Gastroenterology.

[70] Gyawali C.P. (2021). Radiofrequency ablation for gastroesophageal reflux disease: a review. Gastroenterol Hepatol.

[71] Vela M.F. (2022). Endoscopic fundoplication techniques for GERD: a systematic review. Gastroenterology.

[72] Pandolfino J.E. (2020). Acupuncture and moxibustion in the treatment of gastroesophageal reflux disease: a review. J Neurogastroenterol Motility.

[73] Mittal R.K. (2021). Combination therapy of Western medicine and traditional Chinese medicine in refractory GERD: a clinical review. Gastroenterology.

[74] Lu M., Jieyu S. (2022). Capsules in the treatment of refractory GERD: a randomized controlled trial. J Traditional Chinese Medicine.

[75] Gyawali C.P. (2023). Warm acupuncture and moxibustion in the management of refractory GERD: a clinical review. Gastroenterol Hepatol.

[76] Vela M.F. (2024). Acupoint embedding combined with traditional Chinese medicine for refractory GERD: a clinical study. Gastroenterology.

